# Changes in P3b Latency and Amplitude Reflect Expertise Acquisition in a Football Visuomotor Learning Task

**DOI:** 10.1371/journal.pone.0154021

**Published:** 2016-04-25

**Authors:** Kyle K. Morgan, Phan Luu, Don M. Tucker

**Affiliations:** 1 Electrical Geodesics, Inc., Eugene, OR, United States of America; 2 University of Oregon Department of Psychology, Eugene, OR, United States of America; Vanderbilt University, UNITED STATES

## Abstract

Learning is not a unitary phenomenon. Rather, learning progresses through stages, with the stages reflecting different challenges that require the support of specific cognitive processes that reflect the functions of different brain networks. A theory of general learning proposes that learning can be divided into early and late stages controlled by corticolimbic networks located in frontal and posterior brain regions, respectively. Recent human studies using dense-array EEG (dEEG) support these results by showing progressive increases in P3b amplitude (an Event Related Potential with estimated sources in posterior cingulate cortex and hippocampus) as participants acquire a new visuomotor skill. In the present study, the P3b was used to track the learning and performance of participants as they identify defensive football formations and make an appropriate response. Participants acquired the task over three days, and P3b latency and amplitude significantly changed when participants learned the task. As participants demonstrated further proficiency with extensive training, amplitude and latency changes in the P3b continued to closely mirror performance improvements. Source localization results across all days suggest that an important source generator of the P3b is located in the posterior cingulate cortex. Results from the study support prior findings and further suggest that the careful analysis of covert learning mechanisms and their underlying electrical signatures are a robust index of task competency.

## Introduction

As a person transitions from being a novice to expertly performing a task, the neural processes initially used to acquire and perform the task disengage; allowing more cognitive resources to be available for other functions. [1] The path to becoming an expert involves multiple learning stages, but can be simplified into two distinct stages: early and late, where contrasting processes define each stage. The early stages of learning are defined by a reliance on controlled processes, which require a person to be actively attentive, and are limited by working memory capacity. [1] In contrast, the later stages are defined by its lack of reliance on controlled processes, reflected as automated performance, and are not limited by working memory capacity and can be carried out subconsciously under the right context. [1]

Modern imaging evidence have delineated distinct brain networks that are involved in these two stages of learning. [2] The frontal lobe is responsible for the executive monitoring of unfamiliar stimuli; a process that is integral to the early stages of learning. [2][3] By contrast, cortical regions in the posterior corticolimbic system are engaged when subjects demonstrate proficient performance in the late stages of learning. [2][3] These posterior corticolimbic structures, which include the hippocampus and posterior cingulate cortex (PCC), consolidate information and, with sufficient practice, enable performance to be more automated, removing the need for executive control.

The P300 Event Related Potential (ERP) obtained through Electroencephalography (EEG) is of particular interest for tracking the onset of the late learning stage and the development of expert cognition. Because the P300 can vary in its topographic distribution as well as the conditions under which it is evoked, it is now recognized that there is a family of P300 components. [4] The P3a, which has a mediofrontal scalp distribution, is commonly evoked during a 3-stimulus oddball task when participants are exposed to infrequent, novel (non-target) stimuli. [5][6][7] Of more relevance to the current study is the P3b, which is traditionally found over more parietal scalp sites, and occurs within the same oddball task but in response to stimuli that require an action (such as a response or silent count). [7]

Conventionally, the P3a is thought to reflect the attentional shift caused by the mismatch between a novel stimulus in a series of expected stimuli, whereas the P3b reflects the match between a stimulus and the voluntarily sustained attentional trace. [8] However, this popular theory for the P3b and voluntary attention cannot fully explain the results of several previous studies which showed a linear increase in P3b amplitude coinciding with the acquisition of a response mapping to the point of expert performance. [9][10][11][12][13] It is generally accepted that attention decreases with expertise, and thus if the P3b were a reflection of controlled attention a decrease in amplitude when participants approach expert performance is expected. [14][15][16][17] Results from our previous studies, wherein P3b amplitude continued to increase as participants transitioned from novice to more automated performance, is more consistent with the context updating theory of the the P3b. [18][19]

Under context-updating theory, the P3b indexes the updating and/or confirmation of the context under which an action is learned and performed on a trial-by-trial basis. [7][9][18] The context can be information pertaining to the rules of a task, or even the environment under which knowledge was acquired. Relevant to the dual-stage model of learning, the early stage aids in the formation of this context (not indexed by the P3b) and the posterior corticolimbic system maintains it. Once the context is formed, the constant maintenance and recall of this information helps to guide a person toward selecting the correct action in response to a stimulus quickly and efficiently. The P3b reflects the time-course by which the context is updated and the processing resources that were available when the context was referenced. [19] The sources of the P3b remains to be defnitively resolved. However, results from our source localization studies as well as data from human intracranial EEG (icEEG) and animal studies revealed common P3b sources: the parietal lobe, PCC, medial temporal lobe, and superior temporal sulcus. [20][21][22][23][24][25][26][27]

Previously we performed three dense-array EEG (dEEG) studies focused on the dual-stage theory of learning using an arbitrary visuomotor association task. [19][28][29] In these studies, we used a Go/No-Go task that required participants to learn arbitrary visuomotor associations to form an appropriate action. [30] The participants were tasked with associating a simple visual stimulus (numbers) with a specific button press on a key pad. Our results demonstrated that increases in P3b amplitude reflected performance improvements as participants achieved task proficiency and reached behavioral automaticity in the late learning stage. However, these previous studies utilized very simple stimuli that may not accurately reflect the crucial contextual parameters under which the majority of learning is accomplished in the real world.

The goal of the present study was to confirm and extend the previous findings by examining the P3b’s relation to behavioral performance measures across the stages of learning in a more realistic learning task. To accomplish this, we tracked the P3b ERP component as our participants were subjected to a multi-day, modified Go/No-Go task that is similar to the cognitive training program used by the varsity football team at the University of Oregon to help new players acquire the playbook. In this task, participants were presented with defensive football formations as viewed from the quarterback’s perspective. Participants were responsible for acquiring the proper stimulus-response mappings that help them determine which defensive formations require input from the quarterback (target formations, or “Go trials”), and which formations do not require any intervention (non-target formations, or “No-Go trials”).

We hypothesized that our participants would be proficient in the task by the end of the first day of training, and that the presence of the P3b would closely mark their initial achieving of the learning criterion. We also hypothesized that the onset of full automated performance and cognition would occur during the first day of training, and that changes in the P3b would parallel the performance improvements that occur during this stage (e.g. reductions in errors and reaction times). With further training in the subsequent days, we hypothesized that the P3b would continue to track performance after the task became well learned, demonstrating that the P3b is a valid measure of realistic visuomotor learning and task performance throughout the late learning stage.

## Methods

### Participants

Fifteen right-handed participants were recruited from the University of Oregon Human Subjects Pool (eight males, seven females), with ages between 18 and 41 years (*M* = 23, sd = 6). All participants had normal or correct-to-normal vision, had no history of head trauma or seizures, and were not consuming medication that could affect their EEG. Participants were pre-screened online for their experience with football in order to reduce the chance of contextual familiarity confounding differences in skill acquisition rate. Only the participants who were comfortable recognizing variations in defensive and offensive football formations (e.g. participants who had a history of playing football, or were an avid fan of the game) were qualified to participate. Before each session, participants provided informed written consent and filled out several mood questionnaires. The mood questionnaires were not used for analysis, but were collected as part of a standard lab procedure in the case that they might be useful if a participant displayed adverse behavior during the study. Data from all participants who completed all 3 days of the study were included in our analyses. The research protocol was approved by the University of Oregon and Electrical Geodesics, Inc. (EGI) institutional review boards, and the study took place in the Brain Electrophysiology Laboratory at EGI.

### Task

**Fig 1:**
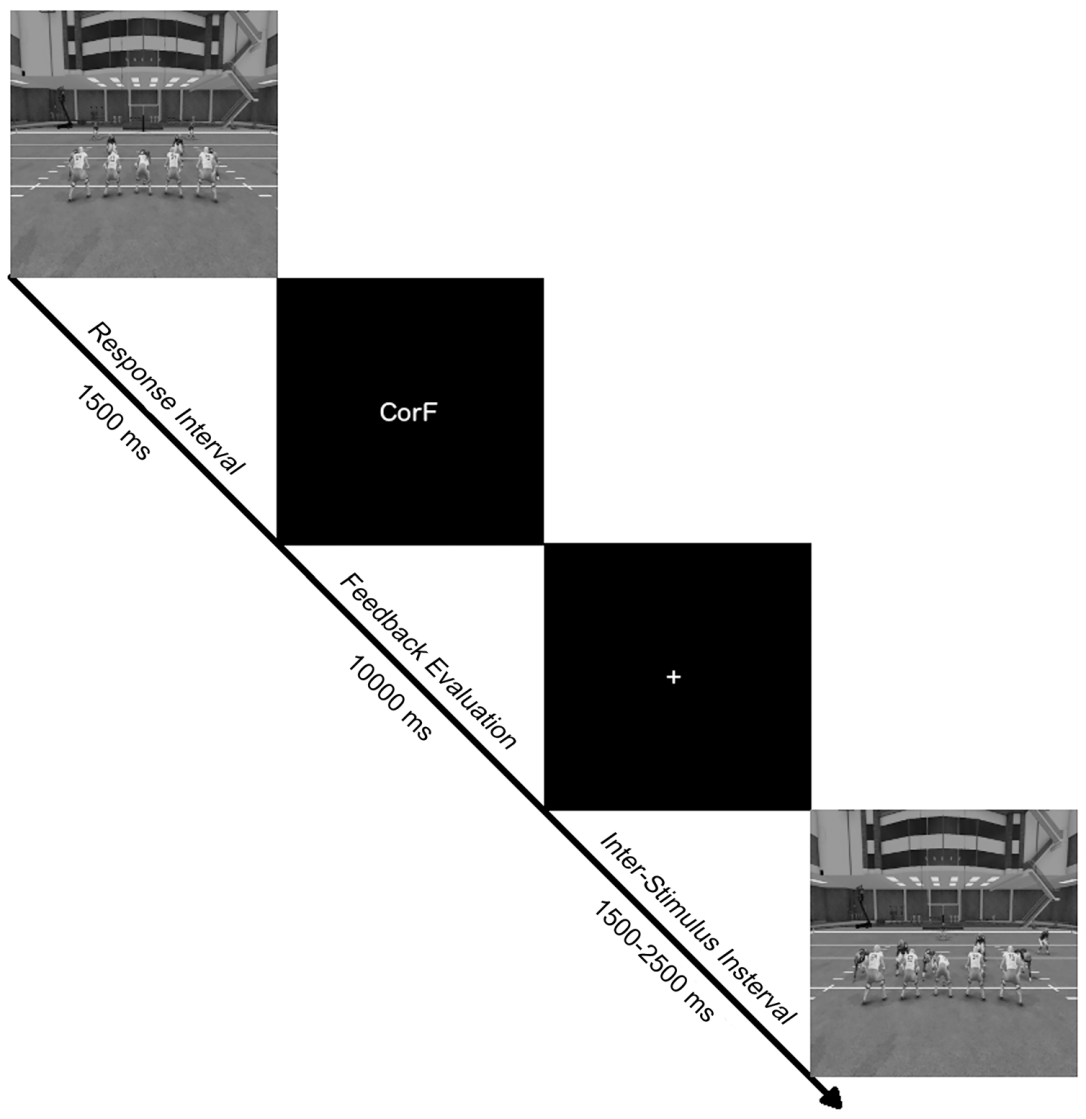
Diagram of events within a single trial. Formations were shown for 1500 ms or until a key-stroke was made. Immediately following a response (or non-response), contingent feedback was shown for 10 s or until the participant made another keystroke. Upon feedback termination, a fixation mark was shown for the duration of the inter-trial interval of 1500–2500 ms before the next formation was presented.

The task used in this study was adapted to resemble the cognitive training program used at the University of Oregon to aid new football recruits in learning the playbook and familiarizing themselves with an opponent’s playing style (Axon Sports, LLC, Phoenix, AZ). Likewise, the paradigm was a modified version of a traditional go/no-go discrimination task. [31] On each trial, 1 of 8 defensive formations were presented centrally on a 43 cm (diagonal) computer monitor for 1500 ms. Half of the formations were randomly selected as “go” stimuli, and the other half were designated as “no-go” stimuli. The formations were presented at random, with the restriction that a formation could not be presented twice in a row. Participants had to either press, or refrain from pressing, a key on a keypad when a formation was presented. For the go stimuli, participants had to learn to respond with the appropriate digit on the correct hand for each stimulus. The participants were given four digits to respond with (digits I and II of both hands), and each of the four go stimuli were mapped onto a specific digit. Each formation was presented on the screen for 1500 ms or until a key-press occurred. Immediately after each trial, specific feedback about performance on that trial was provided, Fig 1.

The feedback given to the participant were designed to provide them all of the information needed to learn the response mapping. In sum, there were six types of feedback: ErrorGo (error of omission), ErrorNG (error of commission), Correct (correct response to a go stimulus but made with the wrong hand), CorH (correct response to a go stimulus with the correct hand but wrong digit), CorNG (correct withholding of a response to a no go stimulus), CorF (correct response to a go stimulus with the correct hand and digit). Feedback were presented for 10 s, or until the participant terminated the feedback with a button press. Upon termination of the feedback, the next trial began between 1500 and 2500 ms later, Fig 1.

To motivate participants to continuously improve on the task, they were made aware that they would be compensated based off of their study performance. To track performance, point values were assigned to each contingent feedback: correct responses (CorNG and CorF) would earn them eight points, whereas errors (ErrorNG and ErrorGo) would lose them eight points. Partly-correct responses (Correct and CorH) would lose a participant four and two points. Participants were informed that they would be able to track their score across each block, and that their final score will determine how much they are compensated on each day. Participants started with a score of zero, and were explicitly told how their point total will be converted into their compensation rate ($15–45).

### Procedure

Following the informed consent process, participants were fitted with a 256-channel HydroCel Geodesic Sensor Net (HCGSN) and placed 55 cm in front of the computer monitor. A chin-rest was used to minimize head movements and keep the distance to the monitor fixed. Participants were explicitly told that there were 8 defensive formations in this study, and that they must learn which formations require a specific key stroke and which formations require them to make no response at all. To add relevant context to the learning environment, the act of pressing a button corresponded to the quarterback’s decision to “hike” the football in response to a target formation. Similarly, an inaction corresponded to the quarterback’s decision to not hike the ball, and instead could be assimilated to halting gameplay (e.g. calling a timeout, or pausing to change a play at the last second). The response feedback that would help teach the participant to make the correct decision were explained clearly on a piece of paper, and participants were allowed to look over the feedback for several minutes.

Once the participant could demonstrate an understanding of the feedback to the research assistant, a short practice block consisting of 30 trials followed. Formations used in the practice block were not used in the actual experimental blocks. For the experiment, 8 blocks of 100 stimuli (800 trials per session) were used. Each participant underwent 3 training sessions, and each session was scheduled exactly 48 hours apart within the same week (Monday, Wednesday, and Friday). The practice block was only given during the first session, and on average each session lasted around 2.5 H. All participants displayed proficiency in the task within the first session, and were compensated an average of $40 for each session.

### Learning Criterion

To simplify the analysis process, we used the fixed-number of consecutive responses method (FCCR) in order to determine when a participant had sufficiently acquired the response mapping as we have done in the past. [28] With this method, a subject fulfilled the learning criterion when they could make four correct responses (or non-responses) in a row for each stimulus. Because the time before this learning criterion was met is a period where participants could not differentiate between whether they needed to withhold or make a correct hand-finger response for a given stimulus, all trials preceding the fulfillment of this criterion were included in a “pre-learning” condition (this includes all trials where errors were committed, for both Go and No-go stimuli). However, because we are only concerned with how a subject acquires and demonstrates a *response mapping* and not *response inhibition*, only the go-trials where the participant provided a fully correct response (CorF) were included in a “post-learning” condition after the learning criterion was fulfilled.

### EEG Recording and Post-Processing

The dEEG was recorded using a 256-channel HydroCel Geodesic Sensor Net and the data were amplified using a Net Amps 400 Amplifier (Electrical Geodesics, Inc., Eugene, OR). Recordings were referenced to Cz and impedances were maintained below 50 kΩ. dEEG was bandpass filtered (0.1–100 Hz) upon being sampled at 250 s/s with a 16-bit analog-to-digital converter.

After recording, signals were filtered between .1–30 Hz bandpass and segmented into 1200 ms long segments time-locked to the onset of each stimulus (segments extended 200 ms before and 1000 ms after the stimulus onset). Segments containing eyeblinks, muscle tension, major eye movements, or large head movements with 10 or more channels exceeding an absolute voltage threshold of 140 *μ*V were excluded from a participant’s average. Segments containing minor eye movements (saccades) were not fully rejected given the lack of overlap between the latency and distribution of the saccades with the latency and location of the P3b. All data were re-referenced to the average reference for analysis.

### EEG Source Analysis

Source analysis was performed using GeoSource (version 2.0) software (Electrical Geodesics, Inc., Eugene, OR). The software relies on the MRI and CT scan of a single subject (Colin 27) to construct an atlas model of the brain and head that is used to estimate the sources of scalp EEG. The brain (gray and white matter) and cerebrospinal fluid (CSF) are segmented as they appear in the MRI, whereas the skull and skin surfaces are characterized from the CT. These two volumes are then co-registered together. Once registered, the gray matter tissue is parceled into 7 mm voxels which serve as individual source locations with three orthogonal orientations, resulting in 2,394 triples sources. Following the construction of the head model, averaged 256 sensor-locations are then registered to the scalp surface.

A Finite Difference Method (FDM) is used to compute an estimate of how current propagates from the sources in the cortical gray matter to the scalp where EEG is measured. Conductivity values used in the FDM were: 0.25 S/m for the brain, 1.8 S/m for CSF, 0.018 S/m for skull, and 0.44 S/m for scalp. [32] The local autoregressive average (LAURA) constraint was used to compute inverse source estimates. [33]

## Results

### Behavioral

#### Learning effects (trials to learn)

**Fig 2:**
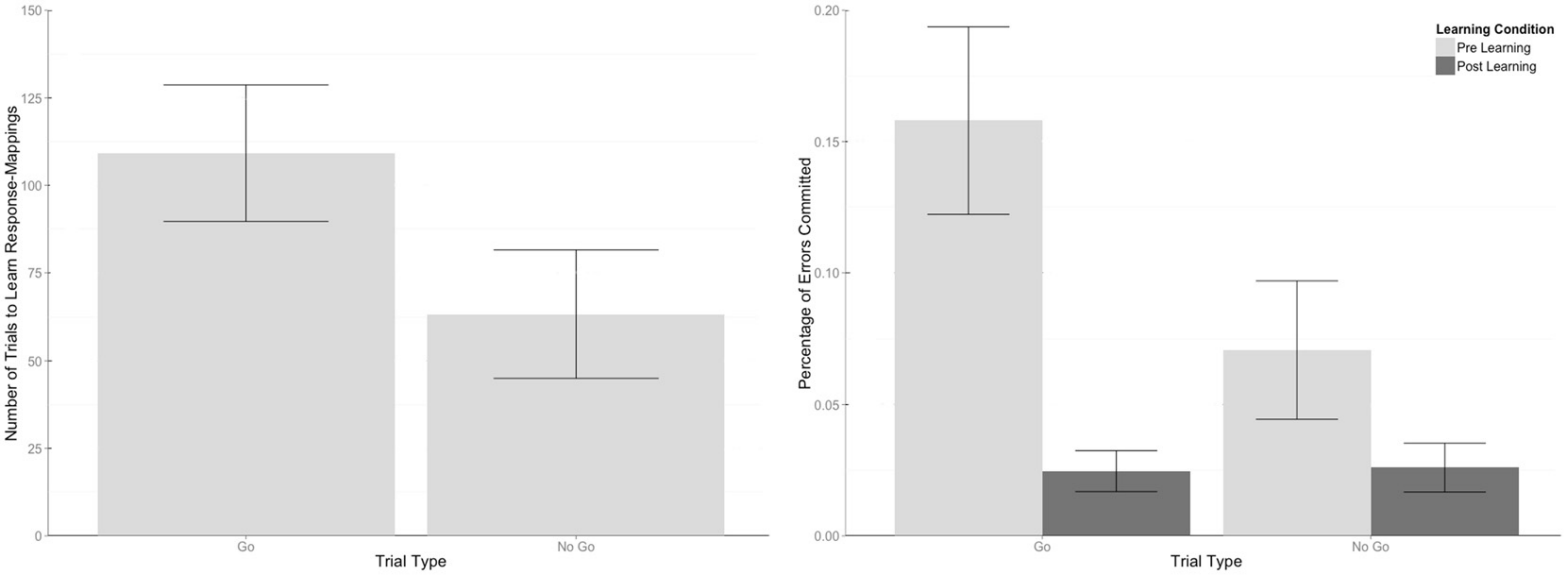
Graphs of trials to learn by trial type and error rate by trial type and learning condition. (Left) On average, Go stimuli took significantly longer to learn than No Go stimuli. (Right) Error rates for both types of stimuli significantly decreased after the learning criterion was met. However, participants made more errors with Go stimuli than No Go stimuli during the learning period. Once the learning criterion was met, there were no differences in the number of errors committed between the stimulus types.

We refer to “learning effects” as effects occurring within the learning process during the first session. A paired samples t-test was run on the number of trials it took each participant to learn the response-mappings, separated by stimulus type (“Go” vs “No Go”). A significant effect was found, *t*(14) = 5.3, *p* < .001, such that Go stimuli took longer to acquire than No Go stimuli. A summary of this effect can be found in Fig 2.

#### Learning effects (errors)

For the error rate calculation, an error was quantified as an error of commission for No Go trials, and any response or non-response that was not fully correct for Go trials (i.e. errors of omission, correct responses to a Go stimulus with the incorrect hand, and correct responses to a Go stimulus with the correct hand but incorrect digit). For each participant, errors were counted for the period before and after the learning criterion was met during the first session only, as all participants acquired the task during the first session.

In a repeated measures ANOVA which used trial type and learning condition (pre-learning and post-learning) as within-subject factors, significant main effects of trial type (*F*(1, 14) = 30.72, *p* < .001), and learning condition (*F*(1, 14) = 66.11, *p* < .001) were found. The nature of these effects show that error rates (collapsing across trial type) were significantly lower after the learning criterion was fulfilled, and that more errors were committed for Go stimuli than No Go stimuli before participants acquired the response-mapping, Fig 2. Additionally, a significant interaction between these two factors was found, demonstrating that there was no difference between Go and No Go errors *after* the learning criterion was satisfied, *F*(1, 14) = 35.3, *p* < .001.

#### Training effects (errors)

All participants sufficiently acquired the task during the first half of the first session and did not commit enough errors during sessions 2 and 3 to define a secondary or tertiary learning period. Due to this, we labeled days 2 and 3 as full training sessions throughout all of our analyses, where we assume most correct responses performed during these days were a result of a participant’s knowledge and expertise in the task, and not due to chance as they may have been during the pre-learning period during day 1. We refer to “training effects” as effects occurring after participants satisfied the learning criterion during sessions 1–3, accordingly. When computing training effects, only the post-learning data from day 1 were used for comparison.

**Fig 3:**
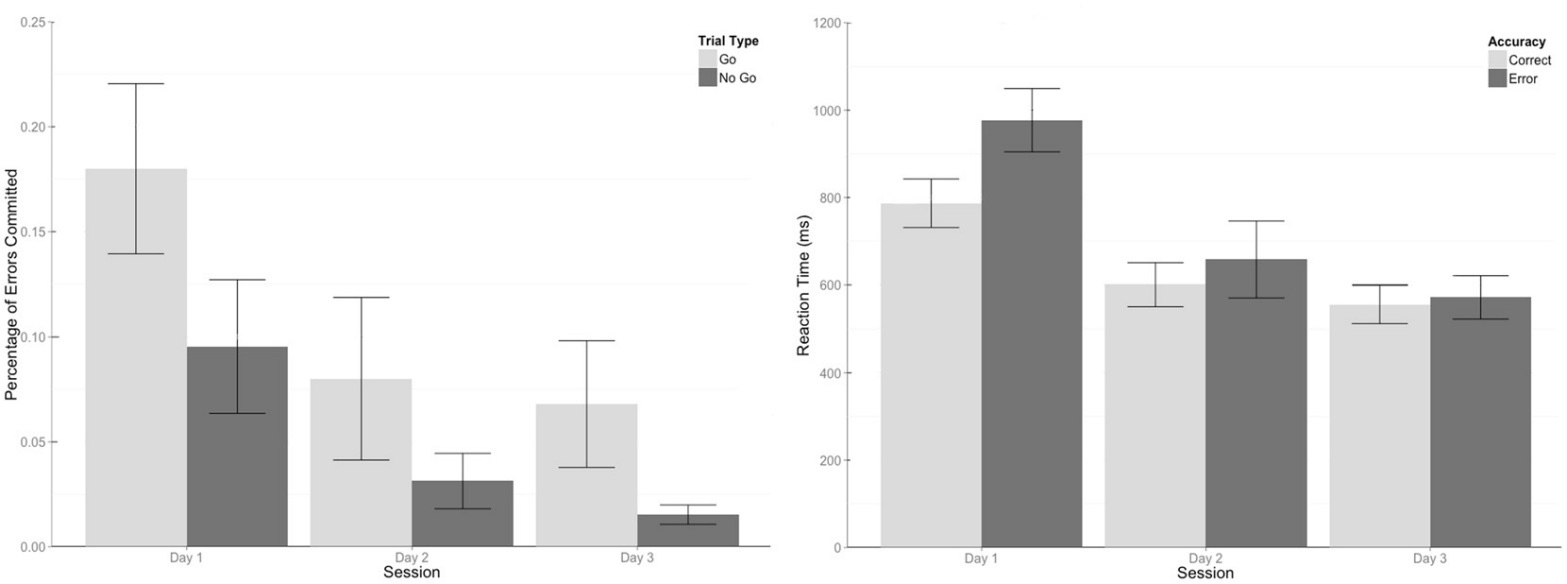
Graphs of error rates and reaction times across training days. (Left) Error rates decreased with practice, and the number of errors were greater for Go stimuli than No Go stimuli for all sessions except for day 2.(Right) Reaction times (RT) decreased with training. In addition, RTs for correct responses were quicker than those for incorrect responses. However, there was only a difference in RT for correct and incorrect responses during the first training session, suggesting that errors made on during this day may differ in nature than those committed during subsequent training days. This interaction corresponds to the session where learning was achieved for all participants.

Trial type and training session (Days 1–3) served as within-subject factors in a repeated measures ANOVA which evaluated error rates across days. Significant main effects for trial type (*F*(1, 14) = 18.74, *p* < .001) and training session (*F*(1.23, 17.18) = 31.49, *p* < .001, Greenhouse-Geisser corrected) were found. The effects show that error rates decreased with practice, and more errors were committed for Go stimuli than No Go stimuli. A significant interaction between trial type and practice session was also found, *F*(1.31, 18.31) = 7.44, *p* = .009 (Greenhouse-Geisser corrected), which showed more errors committed for Go trials compared to No Go trials on days 1 and 3, but no difference on day 2, Fig 3. When collapsing across trial type, significant linear (*F*(1, 14) = 53.955, *p* < .001) and quadratic (*F*(1, 14) = 9.02, *p* = .006) trends were found (Day 1 *M* = 14%, Day 2 *M* = 5.6%, and Day 3 *M* = 4.2%). A comparison of the means shows significant differences in the errors committed on the first day compared to the second and third days (*t*(28) = 6.27, *p* < .001 & *t*(28) = 7.35, *p* < .001, respectively), but no significant difference in errors between the second and third days (*t*(28) = 1.07, *p* = .54).

#### Training effects (RT)

For the reaction time (RT) analysis, RTs for trials where participants made a correct response (CorF) were labeled as “Correct”, and RTs for trials where participants made an incorrect response (errors of commission, correct responses to a Go stimulus committed with the incorrect hand, and correct responses to a Go stimulus committed with the correct hand but incorrect digit) were labeled as “Errors” in an accuracy category.

Accuracy and training session were included as within-subject factors. Significant main effects of accuracy (*F*(1, 14) = 65.77, *p* < .001) and training session (*F*(1, 28) = 164.85, *p* < .001), along with an interaction between the two (*F*(2, 28) = 22.70, *p* = .009) were found. A mean inspection shows that RTs were significantly shorter for correct responses compared to errors (Correct *M* = 647.93, Errors *M* = 735.67). However, RT differences for trial accuracy were only significantly different during the first training session, Fig 3. This interaction suggests that the nature of errors committed during the first training session (where learning occurred) may differ than those which occurred in the remaining training days. Collapsing across accuracy, RTs decrease in a significant linear trend, *F*(1, 14) = 295.72, *p* < .001, Day 1 *M* = 881.88, Day 2 *M* = 629.59, Day 3 *M* = 563.95. Significant differences in all pair-wise comparisons of these RTs were found, Day 1 v. Day 2: *t*(28) = 13.65, *p* < .001, Day 1 v. Day 3: *t*(28) = 17.20, *p* < .001, and Day 2 v. Day 3: *t*(28) = 3.60, *p* = .004.

### dEEG data

**Fig 4:**
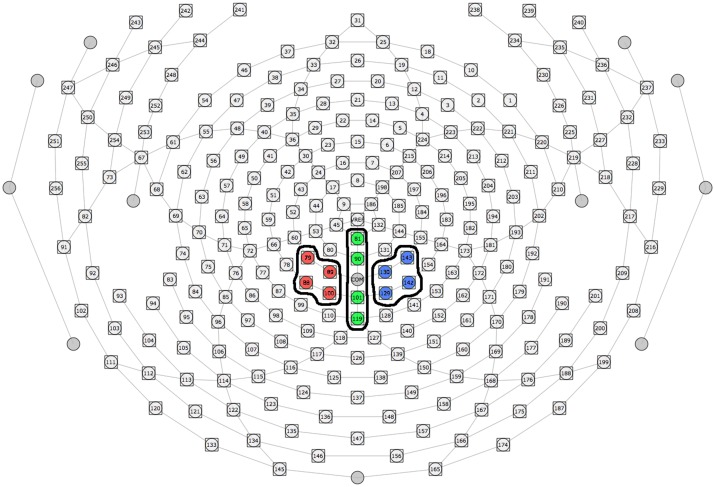
Channel montages used to quantify the P3b ERP component. 3 distinct groups of channels were used to separate the P3b component by laterality (left = red, midline = green, blue = right).

**Fig 5:**
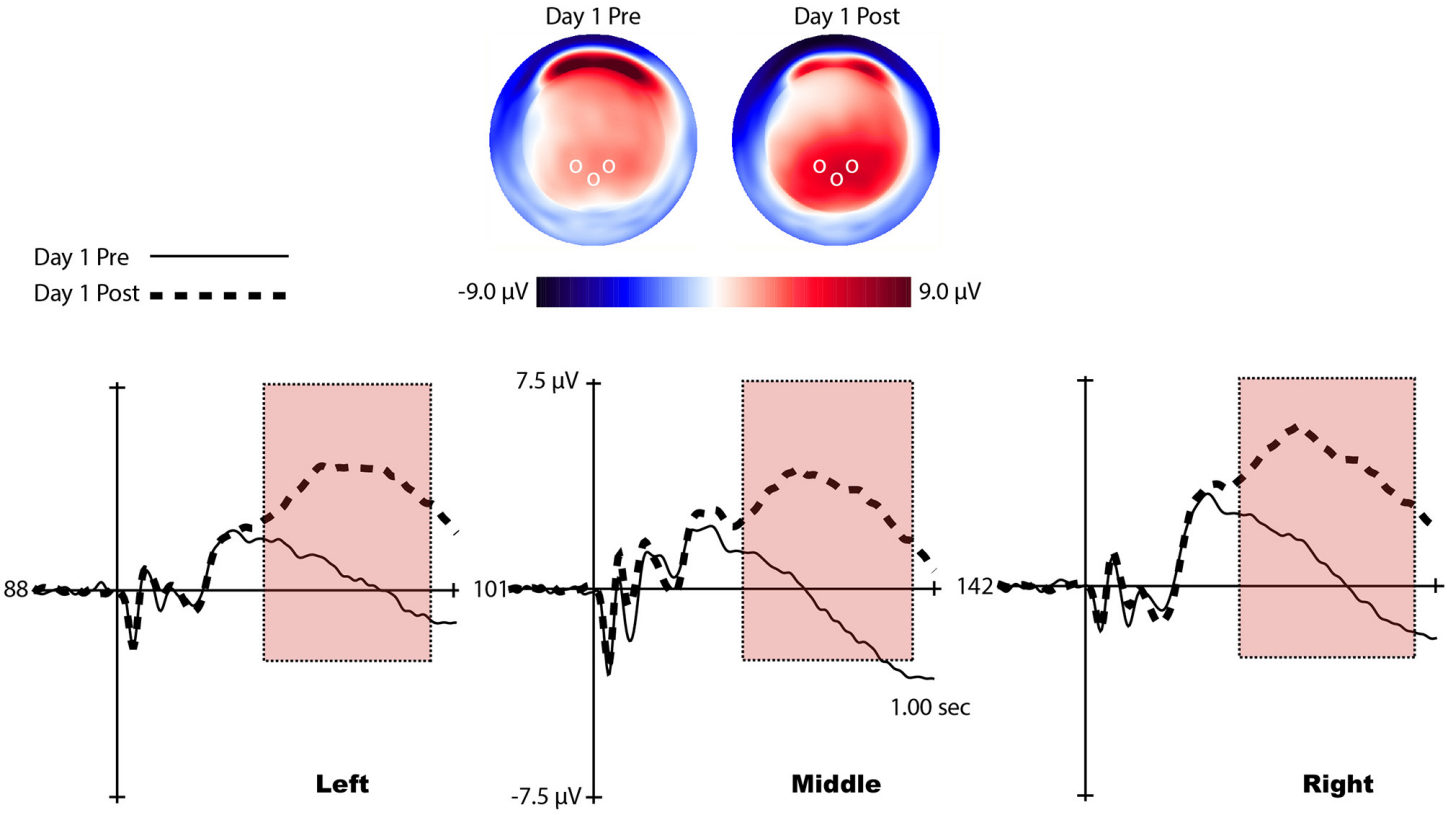
Voltage maps and waveforms of the P3b from representative channels for pre- and post-learning conditions. (Top) Voltage distributions of the P3b across the scalp for both learning conditions. Clear differences in positive energy can be seen around the occipital region. White circles represent the location of the representative channels shown in the bottom of the figure. (Bottom) P3b waveform (red window) displayed by representative channels from each laterality condition. Clear amplitude differences can be seen between learning conditions.

**Fig 6:**
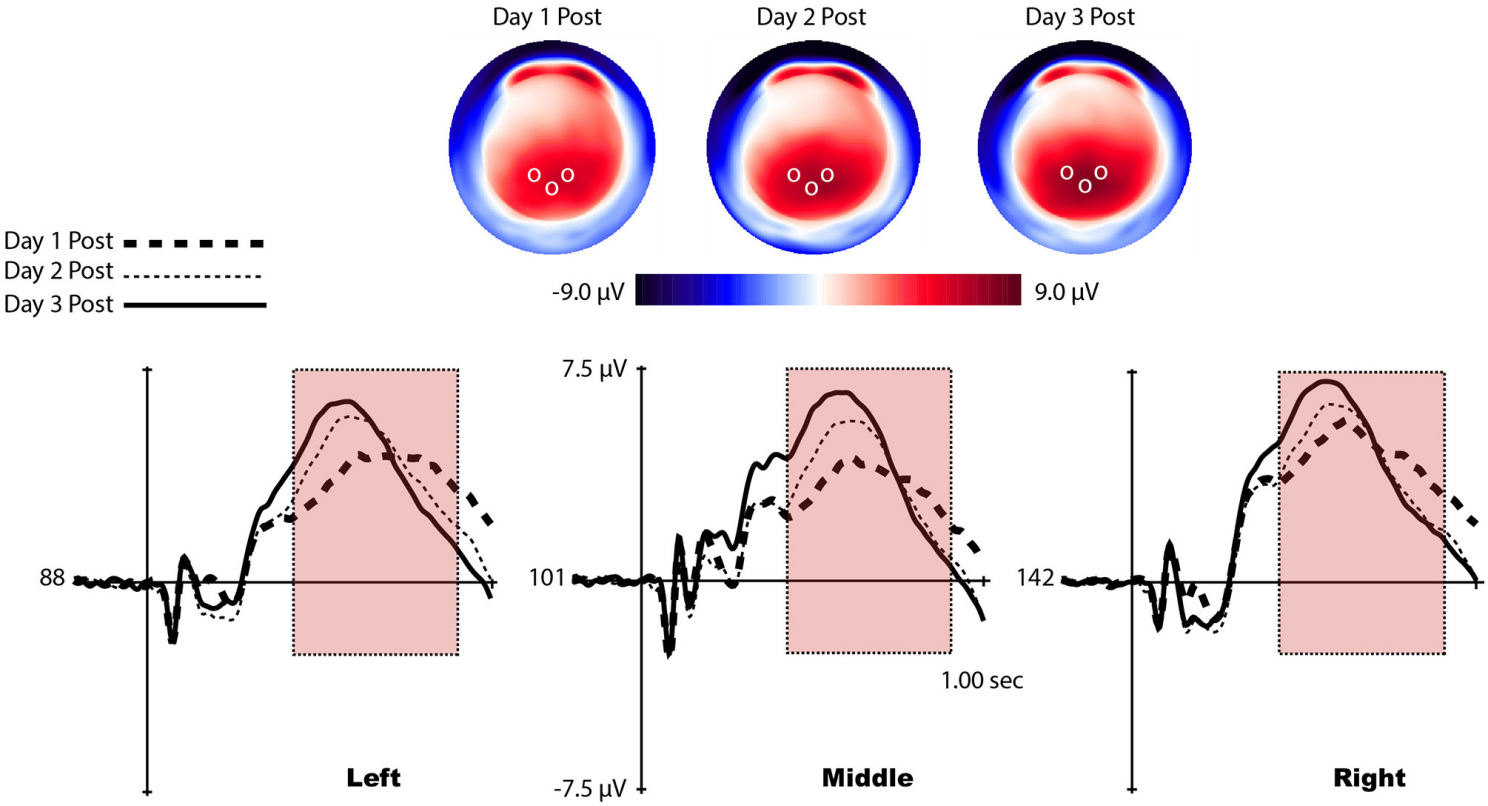
Voltage maps and waveforms of the P3b from representative channels for all training days. (Top) Voltage distributions of the P3b across the scalp for all training days. Clear differences in positive energy can be seen around the occipital region. White circles represent the location of the representative channels shown in the bottom of the figure. (Bottom) P3b waveform (red window) displayed by representative channels from each laterality condition. Clear amplitude differences can be seen across training sessions.

For the P3b analysis, three sets of channels corresponding to laterality (left, midline, and right) were used to evaluate differences in P3b scalp topography based off of similar electrode sites chosen in our previous studies, Fig 4. [28][19] To quantify the P3b, an adaptive mean amplitude corresponding to 22 ms before and after the maximum peak amplitude in a window extending from approximately 450–950 ms after stimulus onset was computed for each separate channel grouping (red windows in Figs 5 and 6). This method was applied to each individual participant and condition, so that small variations in the latency of the P3b were considered.

#### P3b learning effects

A repeated measures ANOVA was performed with laterality (left, middle, and right) and learning condition (pre-learning and post-learning) as within-subjects factors. A significant main effect of learning condition was found, (*F*(1, 14) = 94.43, *p* < .001. No other main effects or interactions were found. The analysis shows that P3b amplitude was much larger after learning had occured, and did not differentiate by hemisphere, Fig 5.

#### P3b training effects (Amplitude)

To evaluate P3b training effects, only post-learning P3b amplitude measurements were used from day 1. Training session (1, 2, and 3) and laterality (left, middle, and right) were included as within-subjects factors. A significant main effect of training session (*F*(2, 28) = 7.35, *p* = .002) was identified, no other main effects or interactions reached statistical significance. A trend analysis reveals a significant linear trend in P3b amplitude with practice when controlling for laterality, *F*(1, 14) = 14.42, *p* < .001. An inspection of our means shows that this trend is positive (Day 1 *M* = 5.63, Day 2 *M* = 6.67, Day 3 *M* = 7.30), Fig 6.

#### P3b training effects (Latency)

Differences in peak P3b latency were computed through identifying the largest positive peak between 450–950 ms after stimulus onset and recording the segment time of the maximum amplitude (red windows in Fig 6). Peak latency was not computed for the pre-learning P3b waveform during the first session because the shape of the P3b did not present a reliable “peak” to accurately perform the analysis. Instead, peak latency was found for the post-learning condition on session 1 and the subsequent training days.

Laterality and training session served as within-subject factors. A significant main effect of training session was found, *F*(1.08, 15.15) = 9.93, *p* = .006 (Greenhouse-Geisser corrected). No other main effects or interactions reached significance, suggesting that P3b latency did not differ as a function of topography. Our training session effect shows a decrease in P3b peak latency with training (Day 1 *M* = 703.22, Day 2 *M* = 620.53, Day 3 *M* = 585.91), Fig 6. A trend analysis reveals that our latency decreased in a significant linear fashion, *F*(1, 14) = 18.87, *p* < .001.

### P3b correlations to behavior

Reaction times in our study appeared to provide the most convincing evidence of when a participant achieved expertise in the task among all other behavioral measures. Large decreases in RT were observed when a participant fulfilled the learning criterion, and they continued to decrease slowly with practice. Additionally, RTs provided a convincing parallel to the decreases in errors across training days, which is the most commonly used measure of task performance. Given the reliability of RT, we focus on correlating our electrophysiological data with RT only.

#### Development of automaticity (RT/P3b Latency Ratio)

The ratio of RT to the peak latency of the P3b (RT/P3b latency ratio) has been used as a measure of automated cognition. [9][34][35] Traditionally, the latency of the peak of the P3b is used to measure the amount of time a participant took to evaluate a stimulus, whereas their reaction time is a combined measure of how long it takes for the participant to evaluate, select, and execute a response to that stimulus. A related, alternative interpretation of this measure is consistent with context-updating, where the P3b reflects the updating or confirmation of contextually relevant information surrounding a stimulus. When evaluating changes in the RT/P3b latency ratio over time, significant reductions in this ratio indicate that response selection (RT) is moving closer to the updating or restoration of contexts; a process that closely follows response evaluation. This reduction indicates that responses come to fruition quicker as a result of automated cognition associated with the very late stages of learning. [35] We would expect any significant reductions in the RT/P3b ratio to occur close to the fulfillment of the learning criterion during the initial training session, followed by a stabilization of the ratio across training days if the participants sufficiently acquired the task and reached automated cognition within the predicted time frame.

**Fig 7:**
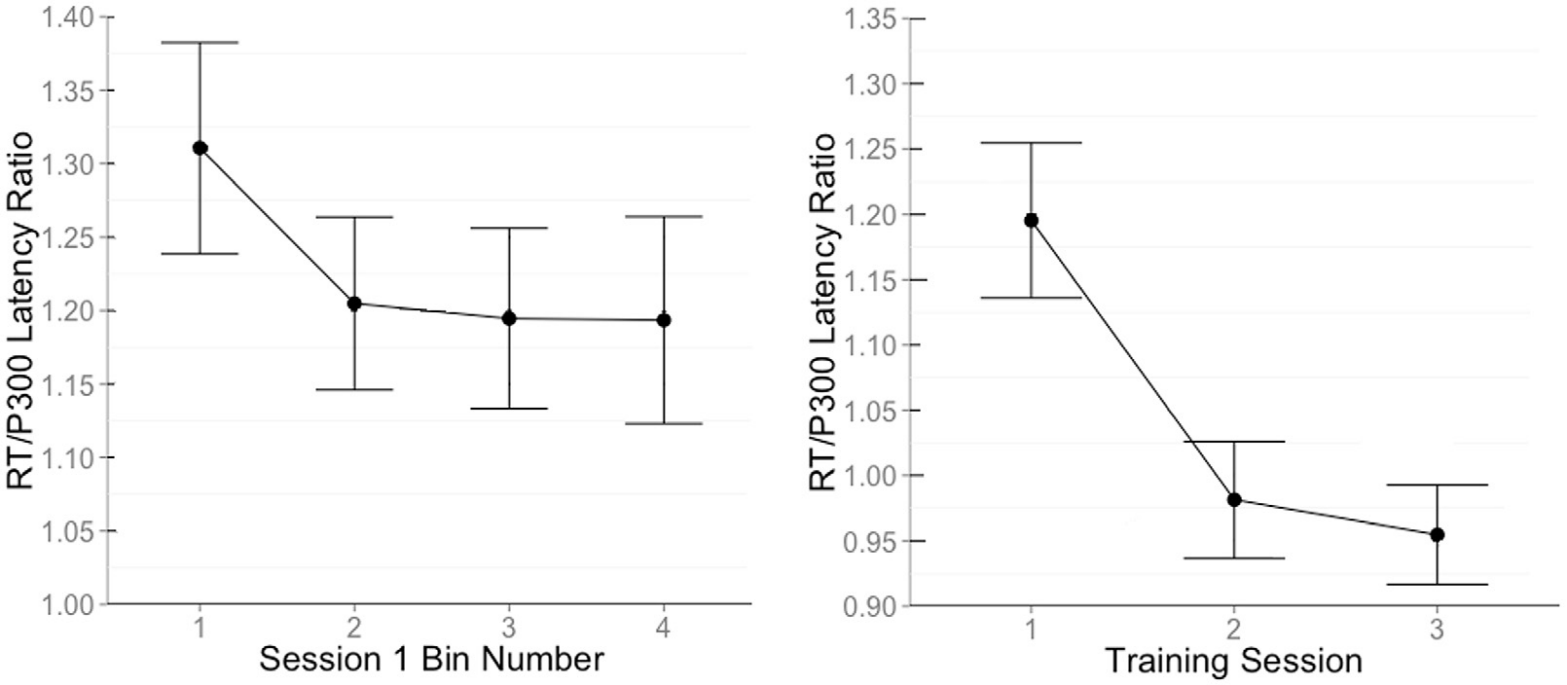
RT/P3b latency ratio plots. (Left) RT/P3b latency ratio displayed in four consecutive bins during the first training session. The ratio decreases as participants progress through the first training session, and then levels-off as they reach the end. (Right) RT/P3b latency ratio for all three training sessions. The RT/P3b latency ratio drops significantly following the end of the first training session, but stabilizes with extended training.

Due to the unreliable nature of interpreting single-trial ERPs, post-learning trials from the first training session were grouped into 4 equal bins for each participant. Separate ERPs were computed for each bin, resulting in 4 average, reliable ERPs per participant. The peak of the P3b was computed by locating the largest positive peak between 450–950 ms after stimulus onset and recording the time of the maximum amplitude. The RT for each bin was then divided by the peak of the P3b for that bin, Fig 7. A significant effect for bin number was found in a repeated-measures ANOVA, *F*(3, 42) = 3.79, *p* = .02. Polynomial contrasts revealed a significant linear trend, *F*(1, 14) = 7.71, *p* = .008, and a pairwise comparison using Tukey’s method shows that there was only a difference in RT/P3b between the first bin and the third and fourth bins (*t*(42) = 2.87, *p* = .04, and *t*(42) = 2.84, *p* = .03, respectively). Results from this analysis suggest that the RT/P3b ratio decreased over time, and then stabilized toward the end of the first training session.

To help us evaluate whether or not automated cognition improved with extended training, the RT/P3b ratio for all post-learning correct responses during the first session were compared to the RT/P3b ratios of the subsequent training sessions. In a repeated-measures ANOVA which included training session as a within-subjects factor, a significant main effect for training session was found, *F*(1.22, 16.03) = 8.62, *p* = .007 (Greenhouse-Geisser corrected). Within this effect, a significant linear trend was discovered, such that the RT/P3b ratio further decreased with extended training, *F*(1, 14) = 15.11, *p* < .001. However, a post-hoc pairwise comparison of means (Tukey’s HSD) showed that the biggest drops in RT/P3b ratio can be seen when comparing the first training session to the second and third (*t*(28) = 3.21, *p* = .009, and *t*(28) = 3.89, *p* = .002, respectively). No significant difference between the RT/P3b latency ratio between session 2 and 3 were found, which suggests the development of automated cognition on the task peaked following the initial training session, Fig 7.

### dEEG source localization

#### Learning effects

**Fig 8:**
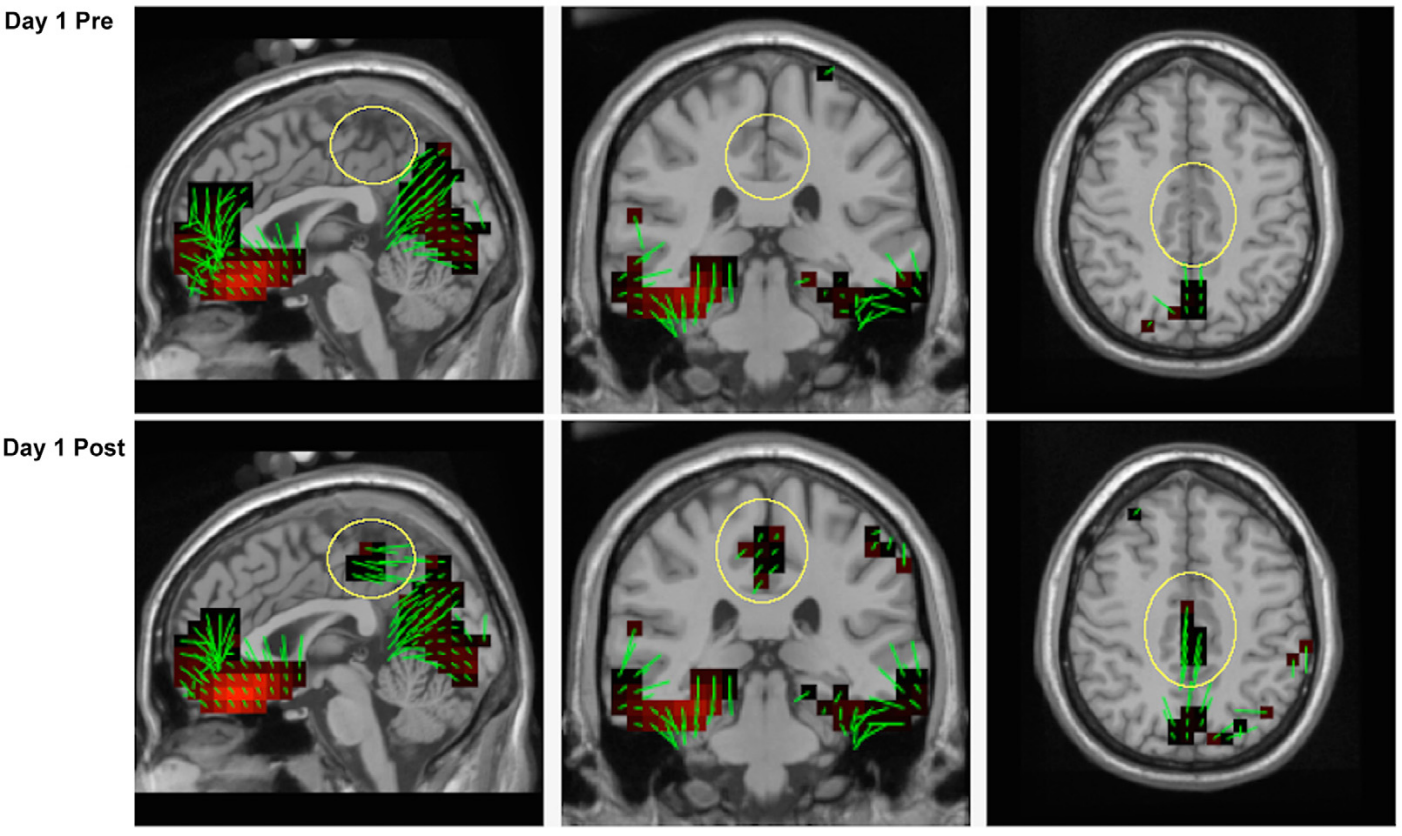
Source estimates for the P3b during the learning session. Activity in posterior cingulate cortex (yellow circles) is absent during the period before participants acquired the response mappings, and is visible immediately after. Green lines at each voxel correspond to orientation vectors pointing in the positive direction.

Source localization was performed using the LAURA constraint and a regularization constant of 10^−3^ on grand-averaged data of all 15 participants. Sources were obtained for the timepoint displaying the largest P3b amplitude for both pre- and post-learning conditions for day 1 (585 ms), Fig 7. Our analysis suggests sources of the P3b in Cuneus and Precuneus (BA7), Lingual Gyrus (BA18), and Fusiform Gyrus (BA37) for the pre-learning condition, and similar sources in the post-learning condition with the addition of Cingulate Gyrus (BA31) and Posterior Cingulate Cortex (BA23). The general absence of cingulate cortex activity in the pre-learning condition is important, as it reflects the lack of P3b presence during the early learning stage (yellow circles, Fig 8).

#### Training effects

**Fig 9:**
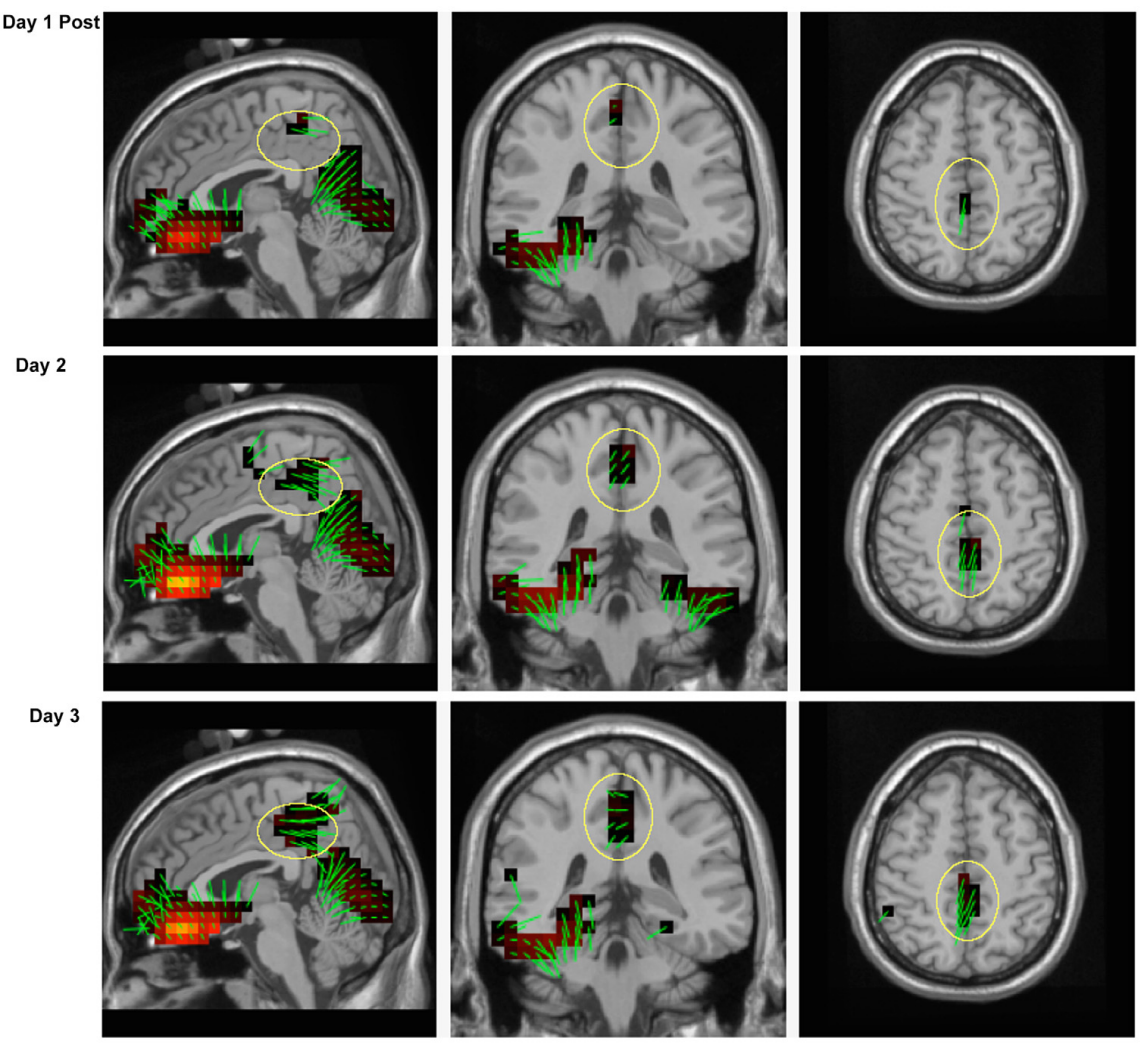
Source estimates for the P3b across training sessions. Activity in posterior cingulate cortex (yellow circles) increases with training.

The post-learning P3b derived from the first training session was used in comparison to the P3b’s obtained throughout the entirety of the subsequent training days. Sources of the maximum peak of the P3b (585 ms) are displayed in Fig 9. All sources overlap with those found in Fig 8, however the amount of cingulate cortex activity appears to differ as a function of training day and P3b amplitude (yellow circles, Fig 9). Specifically, the PCC demonstrates greater engagement with practice, whereas the remaining sources do not reflect this increase.

## Discussion

The main goals of this study were to confirm and extend the relation between the P3b and learning of a realistic visuomotor learning task, in addition to framing the results within the dual model of learning and development of expertise. The results showed that participants learned the task, and their performance continued to improve with training. As participants’ performance improved, P3b amplitude and latency closely tracked the improvements. Both behavioral and P3b measures show a clear break in the rate of change between the first and second sessions and differences between the second and third sessions were less pronounced, suggesting a qualitative shift in the underlying cognitive and brain process involved in learning the task. These results are consistent with a shift from early to late learning systems. Particularly important are the P3b measures and source estimation results, which are consistent with brain regions that have been shown to be engaged during late learning stages.

### Learning and practice

The Go stimuli took significantly longer for our participants to acquire compared to No Go stimuli. This result can be explained by the fact that there were more ways to make an incorrect response for Go stimuli (4) compared to No Go (1). The larger variety of errors for Go compared to No Go can also explain the large margin of errors seen for the Go Stimuli over No Go stimuli in the initial training phase (pre-learning condition from session 1). However, once participants fulfilled the learning criterion, the difference in errors between our stimulus types diminished. This interaction is indicative of a successful acquisition of visuomotor associations.

The dual stage model of learning is supported by the behavioral data. Specifically, the magnitude of error rate and RT reductions between the first and second sessions and the much smaller difference in these two behavioral measures between the second and third sessions suggest a transition between early and late learning systems occurred. [1][2][3] Unlike error rates, which showed no statistical difference between the second and third sessions, a significant RT reduction was observed during this interval. During the first training session, incorrect response RTs were significantly longer than fully correct response RTs. This difference decreased with training, as shown by a statistically significant training session x accuracy interaction. This implies that the nature of errors committed on the first day, when participants were learning the rules of the task, were not the same as those committed in the extended training days. The early learning stage is a time where controlled cognitive processing is most prominent, reflecting the trial-and-error strategy associated with early learning. [36] The shorter RTs associated with errors after learning suggest that a different mode of performance is engaged, being supported by the later learning system. Late learning stage errors, in the present task, likely reflects impulsive responses because they are associated with faster RTs than correct responses, even though this difference did not reach statistical significance.

### P3b and correlations to behavior

Consistent with our previous results, P3b amplitude increased with learning and extended practice. [19][28][29] In other studies, such as that performed by Barcelo and colleagues, P3b amplitude was found to increase with performance on a Wisconsin card sorting task and decreased whenever participants were required to learn a new rule. [37] We hypothesize that the amplitude of the P3b observed in the current study and in previous studies is consistent with the dual stage learning model. [9] Under this model, we propose that the P3b reflects activity of a cortical network which forms a representation of an environmental context that is consolidated with practice, and the involvement of this network increases in the later learning stage in order to help select actions based on an action context. [7][9][29]

In the first session when the early learning system is expected to be strongly engaged, the P3b was not apparent until participants demonstrated that the task was acquired. In a previous study we showed a large P3b that is time-locked to the onset of the feedback in the early learning stage and an absence of the stimulus-locked P3b. Once participants acquired our previous task, the feedback-locked P3b diminished, and was followed by the presence of a stimulus-locked P3b. [38] In the present study, even with the appearance of the P3b in the first session after learning, the dramatic P3b amplitude increase as well as the reduced latency observed with continued training suggest that the late learning system becomes progressively more engaged, Fig 6. In the present study we infer that, when the context was formed during the transition to the late learning stage, the stimuli themselves became a part of the context representation such that actions are now supported as part of the context. The amplitude of the P3b indexes the integration of context and action, whereas the latency refers to the speed at which this evaluative process takes place. [9][38]

The process of context updating, wherein action is integrated to be part of the context, helps us interpret the observed reduction of the RT/P3b latency ratio. In the present study, RT/P3b latency ratio was found to decrease sharply with training and gradually stabilized with extended practice. It has been shown that RT is dissociable from P3b latencty, with P3b latency being indicative of evaluation speed whereas RT is the behavioral output affected by multiple cognitive processes. [9] The RT/P3b latency ratio decrease with training suggests that the action is now more closely integrated with the contextual representation, requiring less involvement of other cognitive or brain processes, Fig 7.

### P3b sources

Previously, sources of the P3b have been localized to ventrolateral prefrontal cortex, posterior parietal cortex, temporoparietal junction, and inferior temporal cortex using combined fMRI and EEG. [39][40] Although no statistical analyses were run, our source analysis in the present study suggest similar sources for the P3b in the lateral and medial areas of parietal cortex (BA7). In addition, we found sources in Lingual Gyrus (BA18) that are consistent with Positron Emission Tomography (PET) results which showed an increase in Lingual Gyrus and Parahippocampal Gyrus involvement during visuomotor mapping [41]. Our findings of strong cingulate gyrus and posterior cingulate cortex sources (BA31 and BA23) overlap with the P3b sources found in the human iEEG literature, non-human vertebrate literature, and our previous attempts at source analysis discussed earlier. [19][20][21][22][23][24][25][26][27][28] An interesting result from this study is the lack of posterior cingulate cortex involvement before learning occurred, but the clear presence of all other sources for the P3b during that time period. As learning developed, and with extended practice, the PCC appeared to increase in activation while the remaining sources were relatively stable (i.e. they did not follow the linear activation pattern shown in the PCC). We interpret this finding as a reflection of the representation and constant updating of action contexts carried out by the posterior dorsal corticolimbic system in the context-updating model of the P3b.

### Limitations

One of the limitations of our study is that EEG only measures cortical activity, whereas there are numerous other studies which suggest several subcortical structures are essential to the learning process. Results from these studies suggest subcortical and cortical systems work synchronously during the early and late learning stages. [41][42][43] Future studies will need to be conducted using methodology that can augment the cortical sources found with the present research (such as fMRI).

Another limitation of the current study is the presence of saccades and other ocular artifacts in the EEG. In order to closely mimic the field of view a quarterback would realistically have on the playing field, the stimuli used in our task were large and caused our participants to horizontally scan each picture for identifying features. The presence of artifacts prevented us from subjecting our data to whole-brain analyses such as Principle Components Analysis (PCA), which would have helped us identify other potential components that correlate with the learning seen in our study. In addition, the artifacts made our frontal components (e.g. the Medial Frontal Negativity) uninterpretable, and forced us to focus on our posterior P3b component only. Although our analysis of the P3b and its sources is important on its own, in the future we plan to reanalyze the results from this study using various signal decomposition techniques in order to combat the ailments present with these artifacts to allow an analysis of frontal activity in the early learning stage.

### Conclusions

The goal of the present study was to investigate the changes in the dorsal posterior corticolimbic system as participants achieve learning and skilled performance in a realistic visuomotor task. By using a task that is relevant to participants’ background and by leveraging information of the brain responses with behavioral measures, we were able to confirm previous findings and extend them to more realistic learning situations. Results from studies that approach more real-world-like tasks, such as the present study, facilitate applications of lab findings to real world applications with more confidence, such as active facilitation of learning and skill acquisition using non-invasive neuromodulation.
